# Inspection Robot Based Mobile Sensing and Power Line Tracking for Smart Grid

**DOI:** 10.3390/s16020250

**Published:** 2016-02-19

**Authors:** Bat-erdene Byambasuren, Donghan Kim, Mandakh Oyun-Erdene, Chinguun Bold, Jargalbaatar Yura

**Affiliations:** 1 Department of Electric Technique, Mongolian University of Science and Technology, Ulaanbaatar 11000, Mongolia; baterdene@must.edu.mn (B.B.); mandakh@must.edu.mn (M.O.-E.); it.chinguun@gmail.com (C.B.); 2 Department of Electronics and Radio Engineering, Kyung Hee University, Yongin-si 446-701, Korea; jargalbaatar@khu.ac.kr

**Keywords:** smart grid, inspection robot, illegal electricity usage, smart sensing, line tracking algorithm, remote detection

## Abstract

Smart sensing and power line tracking is very important in a smart grid system. Illegal electricity usage can be detected by remote current measurement on overhead power lines using an inspection robot. There is a need for accurate detection methods of illegal electricity usage. Stable and correct power line tracking is a very prominent issue. In order to correctly track and make accurate measurements, the swing path of a power line should be previously fitted and predicted by a mathematical function using an inspection robot. After this, the remote inspection robot can follow the power line and measure the current. This paper presents a new power line tracking method using parabolic and circle fitting algorithms for illegal electricity detection. We demonstrate the effectiveness of the proposed tracking method by simulation and experimental results.

## 1. Introduction

One of the aspects that characterizes the development of a society is its energy consumption in all of its forms, where the amount of consumption is steadily increasing. As energy use increases, illegal electricity usage causes an undue additional load on the power distribution system [1]. Therefore, detecting and localizing illegal electricity usage is a significant challenge for power delivery companies in many countries [2,3]. For example, the Minister of Energy and Natural Resources in Turkey has stated that the illegal usage of electricity constitutes 19% of the total electricity consumption in Turkey [4]. The same problem exists in Iran [5], and even Mongolia is now seeking an optimal solution for remote detection of illegal electricity usage [6]. Low voltage power delivery system includes power transformer and subsystems. Electrical voltage supported from the electrical source is decreased because of the power transformer and is transmitted by low voltage transmission line to each of the subsystems. The subsystems include energy users and energy meters. The subsystem then transmits electricity from a note to local users. Illegal electricity usage may exist on the user side of the subsystems, on the outside of nodes or on the low voltage transmission line. Electricity could be used illegally in the four ways. One way is switching of energy cables at the energy meter box. Other ways are use of external phase before the energy meter terminals, use of fixed magnet and use of mechanical objects [7].

A number of detection methods for illegal electricity usage have been researched in recent years. The detection methods of illegal electricity usage are classified into two kinds. These are off-line and real-time methods. Off-line detection methods are based on statistical tools, which use the measurement data of energy users over several days and years. Remote detection methods in real time are classified as follows: Smart meter method, high frequency signal processing method, and inspection robot based methods [8]. Smart meter based remote detection methods are based on the current differences between two energy meters [4]. High frequency signal processing based remote detection method uses a disconnection conception of energy users. There, illegal electricity usage can be detected by measuring the line impedance when the power distribution system of all users are disconnected and then a low-voltage signal having a high frequency component is transmitted to the power distribution system [5]. Recently, inspection robot-based detection systems are widely utilized in power line measurement [9,10,11,12,13,14].

**Figure 1:**
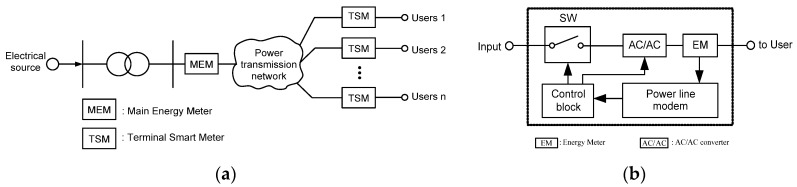
Detection system with inspection robot for illegal electricity usage: (**a**) proposed power delivery system; (**b**) terminal smart meter (TSM) [9].

In this paper, we propose an illegal electricity detection system using a new power line tracking algorithm for mobile sensing based on an inspection robot and smart meters. Figure 1 illustrates an illegal electricity detection system using an inspection robot and smart meters.

**Figure 2:**
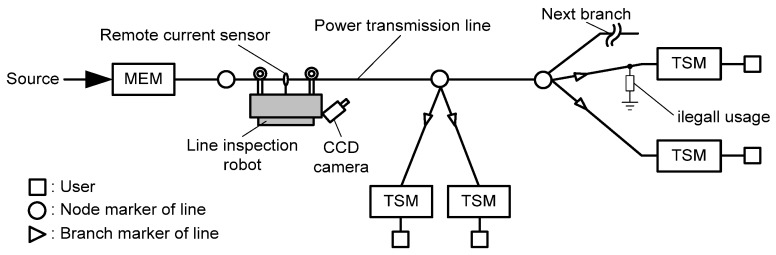
Working environment of inspection robot for detection system [9].

In this detection system, the main energy meter (MEM) and the terminal smart meter (TSM) are installed on the starting node and on the final end of each electrical user, respectively. MEM measures the total energy of all electrical users. At the same time, TSM measures the energy usage of each user and transmits the measured energy value through the power line communication system. If an illegal electricity usage occurs on the power delivery system, the measured value of MEM and the summation of each TSM measurements will not be equivalent. In this case, the detection system begins a process by switching the status of TSMs, as shown in Figure 1b. The switch of a TSM can be either turned on or off by the command of a control block with a high frequency component. The voltage sag is converted to a normal voltage by AC/AC converter, which is created by the switching frequency. If MEM measures some amount of current, even though the user’s currents were switched off by TSMs, that means illegal electricity usage through that power line. But, when a user’s currents are switched on by a TSM, the measured current of the MEM represents the total current of the power distribution system. Therefore, an inspection robot can remotely measure those two kinds of currents. The inspection robot can crawl and move under the transmission line and detect obstacles using camera and other sensors. When the inspection robot detects an obstacle, it can pass through the obstacle [10,11,12,13,14]. Figure 2 shows the working environment of a detection system with an inspection robot.

**Figure 3:**
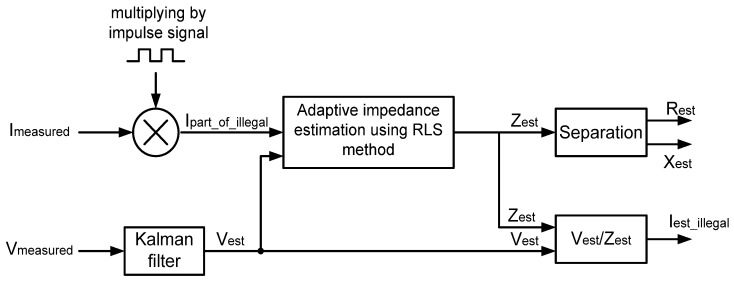
Separation process of illegal current [9].

The inspection robot starts to move away from a MEM and its motion is continued even if an illegal current is measured and detected. To detect and localize of illegal electricity usage, the illegal current should be separated from measured current using the inspection robot. Figure 3 shows the signal process to separate the illegal current.

When the measured current from the inspection robot is multiplied by an impulse signal, the illegal current section can be extracted. In this case, the impulse signal becomes active when users are disconnected by TSMs. In addition, the measured current can be multiplied by another active impulse signal when users are connected by TSMs. In this condition, multiplied current represents the total component of current. Furthermore, the measured voltage from the power delivery system is filtered by a Kalman filter (KF) from the noise of measurement equipment. Because a KF generates efficiency response for sinusoid signals with constant frequency. After that, the adaptive impedance is estimated by values of multiplied illegal current section and filtered voltage using Recursive Least Square (RLS) method. For an estimation, the impedance values of illegal electricity usage should be calculated using measured current and filtered voltage when impulse signal is active. It is assumed that illegal electricity usage is not changed and is constant. Then, calculation of the illegal current is possible using an estimated impedance and filtered voltage.

If the measured current from the inspection robot has an illegal current section, the robot continues its motion towards a node marker. When the robot arrives at the node marker, it detects all of the triangle branch markers and moves to the right-most side of the branch by using its CCD camera. The value of illegal current is calculated on the triangle marker of the passed branch. If the illegal current is detected, the robot continues its motion to pass the branch until arrival at the exact location of illegal electricity usage [9].

Used in this way, the exact location of illegal electricity usage can be transmitted by the robot. After transmission, the robot goes back to the node and checks the next branch.

**Figure 4:**
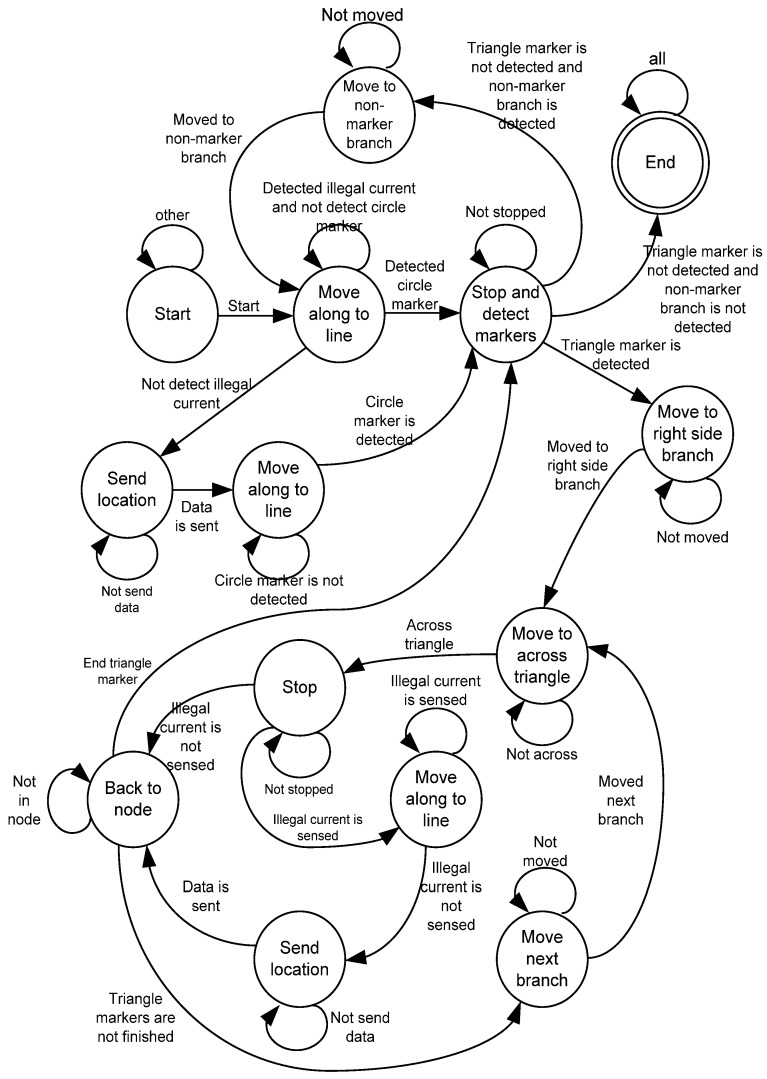
Finite state diagram of inspection robot [9].

If the illegal current is not detected on the triangle marker of the passed branch, the robot goes back to the node and checks the next branch. When all branches with triangle markers are examined, the robot goes to a non-marker branch and repeats its process. The detection process terminates when there is no branch having any markers, which is equivalent to a full coverage inspection of the entire power delivery system. The finite state diagram of the inspection robot is shown in Figure 4.

When the above mentioned environment is used for remote detection of illegal electricity usage, there is a very important aspect of exact tracking on the overhead power line. This exact tracking is the main reason for correct measurement.

In particular, automatic power line detection and tracking are new topics in the world. This paper presents a new power line tracking algorithm for mobile sensing and detection of illegal electricity usage based on an inspection robot.

## 2. Inspection Robot Based Line Tracking Method

In order to remotely detect illegal electricity usage, the exact tracking of electric transmission lines and accurate measurements are very important. To do accurate tracking, it is necessary to save the distances of the remote current sensor and the electric transmission line with a constant value. For the tracking process, the distances of the remote current sensor and the electric transmission line are measured by various types of sensors. For example, there are ultrasonic sensors, proximity sensors, and digital cameras. In this case, the measurement error is generated by sensors, which is inconvenient for the tracking process.

**Figure 5:**
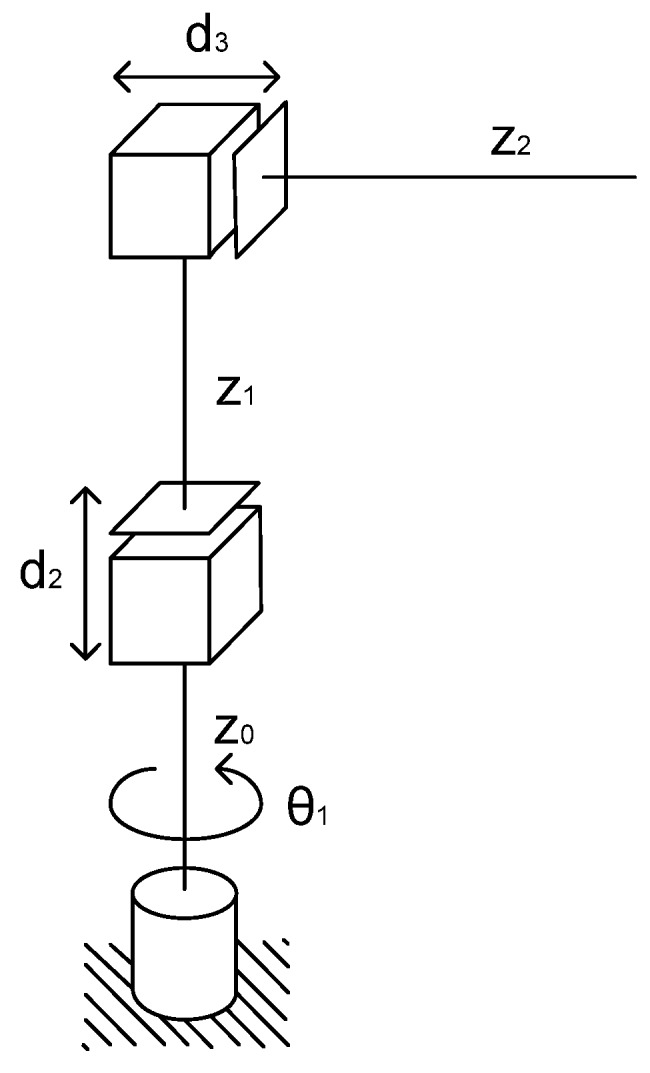
The cylindrical manipulator [15].

The electric transmission line tracking algorithm in this paper consists of several purposes. The tracking algorithm should predict measurement error and correct movement of tracking process for correct current measurement. In order to consider a new tracking method, measurements are executed with the following assumption. We assume that a mobile robot and manipulator are used for the tracking process, the manipulator is placed on the mobile robot. In this tracking system, various type manipulators can be used. However, in this paper, a cylindrical manipulator is selected, which is consistent for movement of tracking electric lines and their swing, and is very easy to use [15]. In addition, it is quick to show simulation results of the proposed tracking algorithm. The cylindrical manipulator is shown in Figure 5.

**Figure 6:**
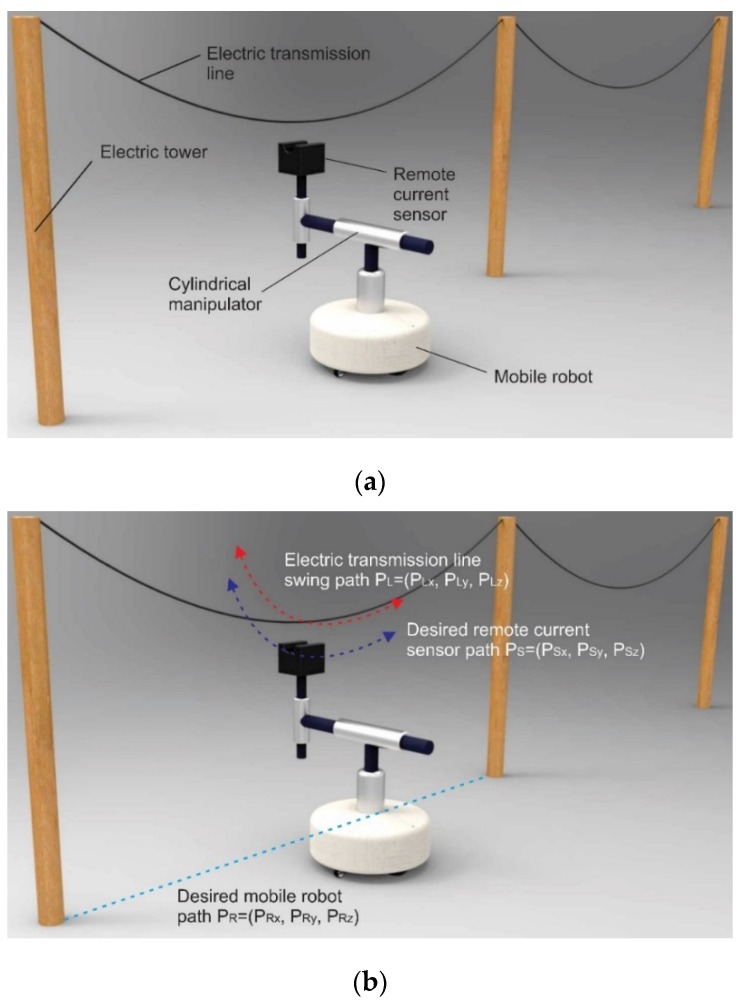
Example of the line tracking system: (**a**) Tracking system collection; (**b**) Traveling paths of the system.

Furthermore, the remote current sensor is placed on the vertical actuator of the cylindrical manipulator, which measures electric current value during the tracking process. The swing of electric transmission lines depends on environmental conditions, temperature and wind speed, and the trajectory is different in such conditions. Figure 6 shows an example of the tracking system described. The tracking system consists of an electric tower, electric transmission line, remote current sensor, distance sensors, cylindrical manipulator, and a mobile robot. The height of the tower for electric transmission lines and the distance between the two towers depends on construction, type of line, voltage level, environmental conditions, and so on. However, the electric wire depends on transmission power and voltage level. The current sensor on the tip of the cylindrical manipulator should be placed near the electric wire with current, where it is possible to remotely measure the electric current [16,17]. In addition, the mobile robot moves along the electric line planned path using several kinds of sensors, and is called an inspection robot [11].

The planned path of the mobile robot, the electric line swing and the desired remote current sensor path are shown in Figure 6b. Distance sensors would be placed on the tip of the cylindrical manipulator, which measures the distance between the current sensor and the electric wire. In addition, PL is the electric line position coordinate, Ps is the remote current sensor position coordinate, and PR is the mobile robot position coordinate. In this system, we assume that:

$$P_{Lz}=P_{Sz}=P_{Rz};\;P_{Ry}=0$$

where: $P_{Lz}$ is the Z coordinate of the electric line; $P_{Sz}$ is the Z coordinate of the remote current sensor; $P_{Rz}$ is the Z coordinate of the mobile robot; and $P_{Ry}$ is the Y coordinate of the mobile robot.

Also, noise is generated in the real system during measurement of P_Lx_ and P_Ly_. Therefore, the input coordinates are:

(1) $${\hat{P}}_{Lx}=P_{Lx}+\delta_{x}$$

(2) $${\hat{P}}_{Ly}=P_{Ly}+\delta_{y}$$

where: $P_{Lx}$ is the X coordinate of the electric line; $P_{Ly}$ is the Y coordinate of the electric line; $\delta_{x}$ is the measurement noise of the X coordinate; $\delta_{y}$ is the measurement noise of the Y coordinate; ${\hat{P}}_{Lx}$ is the X coordinate of the electric line with measurement noise; and ${\hat{P}}_{Ly}$ is the Y coordinate of electric line with measurement noise.

**Figure 7:**
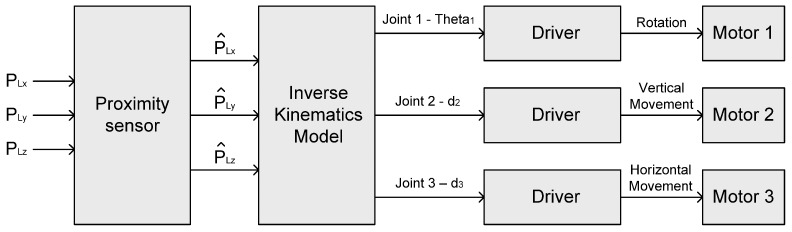
Structure of control system for cylindrical manipulator.

The main objective of the tracking system is to carry out accurate remote current measurements. In order to measure current, when the electric line swings due to wind, the current sensor could follow the electric line. To move to the desired position, the control system for the cylindrical manipulator has to get the coordinate of the movement position from input. Input coordinates are passed to the proximity sensor model. Then, the coordinates with the measurement noise are transferred to the inverse kinematics model. The inverse kinematics model for the control system can calculate the motion angle of the first joint, the displacement of the next two joints and the offsets of joints, and then put in the calculated parameters to the drivers of the motor. Furthermore, the drivers can control each joint motor. The structure of the control system is shown in Figure 7. Motor 1 is installed on the first joint, which can carry out the rotation of the manipulator. However, Motors 2 and 3 are installed on the vertical and horizontal actuator, respectively.

In order to determine parameters of movement, the inverse kinematics equations of the cylindrical manipulator can be used [18]. In our tracking system, the inverse kinematics equations are:

(3) $$\theta_{1}=\text{arctan}\left(\frac{{\hat{P}}_{Ly}}{{\hat{P}}_{Lx}}\right)$$

(4) $$d_{2}=\left({\hat{P}}_{Lz}-Z_{1}\right)$$

(5) $$d_{3}=\sqrt{{\hat{P}}_{Lx}^{2}+{\hat{P}}_{Ly}^{2}}-Z_{2}$$

where: ${\hat{P}}_{Lx},{\hat{P}}_{Ly},{\hat{P}}_{Lz}$ are the electric line coordinates of desired position with measurement noise; $\theta_{1}$ is the angle of Joint 1; $d_{2},d_{3}$ are the joint motions for 2 and 3; $Z_{1},Z_{2}$ are the joint offsets for 1 and 2.

**Figure 8:**
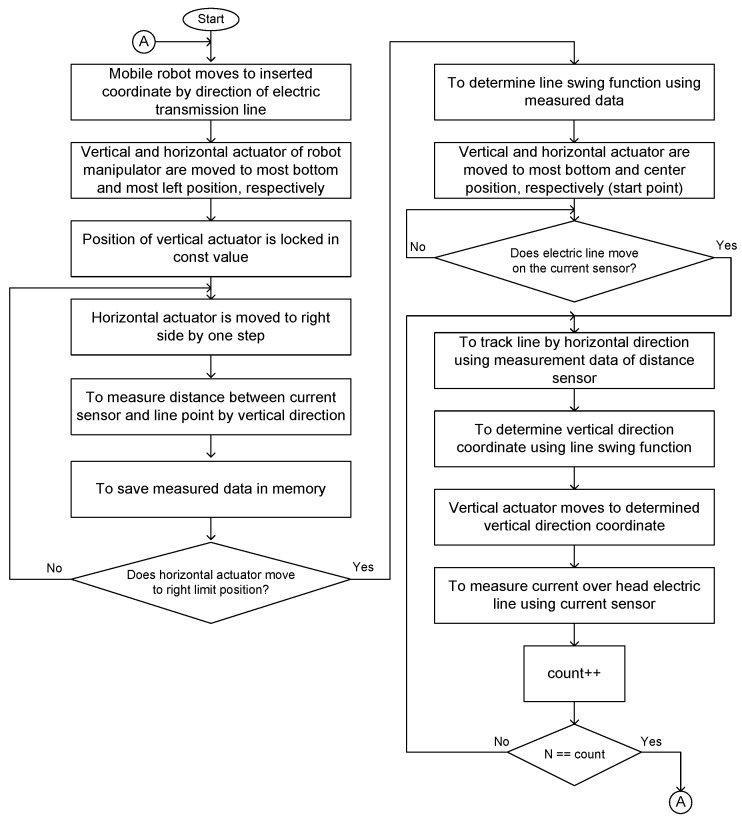
Tracking algorithm of electric line.

Next, the line tracking algorithm is described. The line tracking algorithm is shown in Figure 8. First of all, the mobile robot travels along the electric line and stops at the target coordinate. Then, the vertical actuator movement of the manipulator on the mobile robot is held at a constant value. Furthermore, the horizontal actuator is moved to a horizontal direction. During this horizontal movement, distance measurements between the remote current sensor and the electric line are executed using proximity sensors by vertical direction, and the measurement data is saved. Now, the line swing function can be fitted using gathered measured values, which can represent the swing trajectory of the electric line and movement. The line swing function is mathematical description, which is represents that how conductor of electric line hangs due to wind load. In this algorithm, the fitting process can be implemented by parabolic and circle curve fitting to 2D points. Curve fitting is the process of constructing a curve, or mathematical function, which has the best fit to a series of data points, possibly subject to constraints [19]. The type of curve fits can be divided into three main categories: Least squares curve fits, nonlinear curve fits, and smoothing curve fits.

So, it is necessary to fit a circle to 2D points. Therefore, fitting a circle can be used in this algorithm. Given a set of points ${\left\{\left(x_{i},y_{i}\right)\right\}}_{i=1}^{m}$, m ≥ 3, fit them with a circle ${\left(x-a\right)}^{2}+{\left(y-b\right)}^{2}=r^{2}$ where (a, b) is the circle center and r is the circle radius [20].

**Figure 9:**
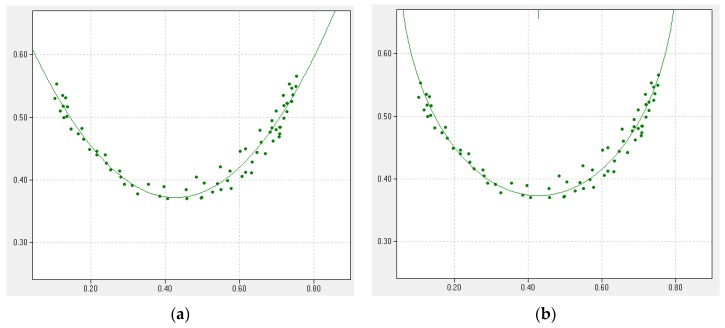
Example of (**a**) parabolic fitting and (**b**) circle curve fitting.

The example graphic of fitting is shown in Figure 9. In this example, the parabolic function is $\;y=ax^{2}+bx+c$, the constants are a = 1.606, b = −1.371, and c = 0.664. However, the circle function is$\;{\left(x-a\right)}^{2}+{\left(y-b\right)}^{2}=r^{2}$, the constants are a = 0.427, b = 0.750 and r = 0.377. In proposed tracking algorithm, parabolic curve fitting is used. It is consistent for electric line swing trajectory of our simulation model.

After fitting, the vertical and horizontal actuator of the manipulator should be moved to a center place, and the system waits for the electric line to get to a vertical direction. If the electric line comes on, the current sensor on the manipulator on the mobile robot starts to track the electric line. In this case, the horizontal movement should be controlled by the measurement data of proximity sensors. However, the vertical movement should be controlled by the determined coordinate using the fitted line swing function. The distance between the remote current sensor and the electric line should be held by the minimum permissible constant value. It is possible to determine the vertical coordinate using the horizontal coordinate by the fitted line swing function. Then, the electric line tracking can be executed. After the current measurement, the mobile robot should move to the next target coordinate along the electric line, and repeat the above process.

## 3. Experimental and Simulation Results

### 3.1. Experimental Results

**Figure 10:**
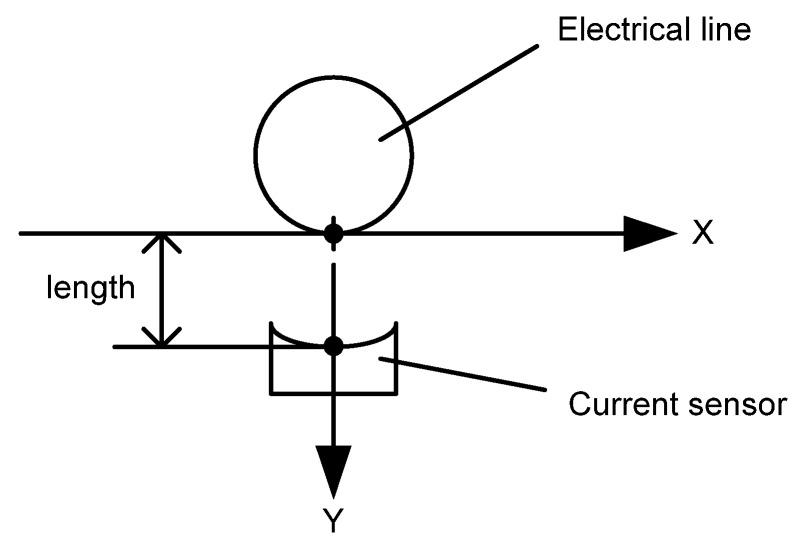
Position of current sensor and electric line for measurement.

The experimental objective is to describe the current intensity, the measurement error depending on distance between the remote current sensor and the electric line. This measurement is very important for determining the minimum permissible distance of measurement. In the experiment, a stationary electric line with load and split core current sensor was used. The type of split core current sensor is CR3100 series, company of CR Magnetics LLC. The cross section, position of current sensor and electric line are shown in Figure 10.

**Figure 11:**
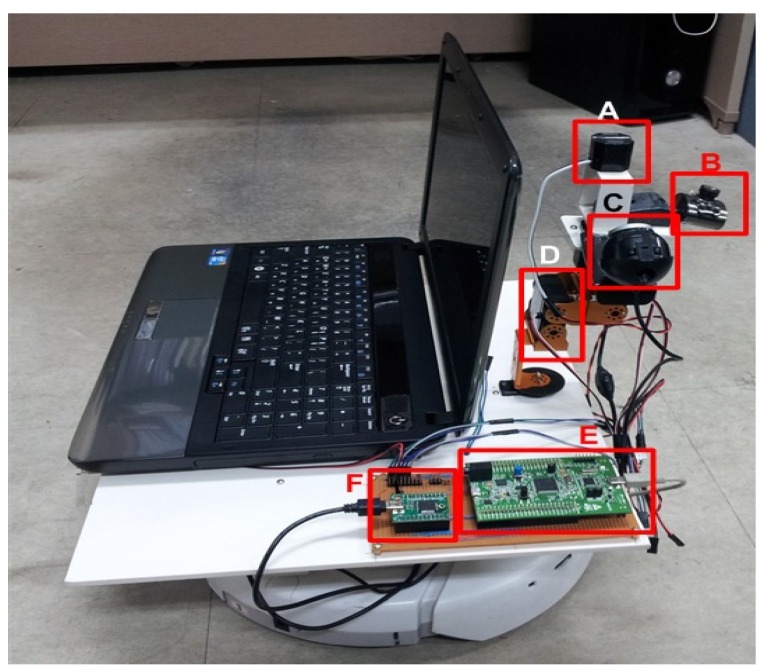
Mobile robot and robot-arm for measurement: (**A**) Split core remote current sensor; (**B**) Image sensor—Camera 1; (**C**) Image sensor—Camera 2; (**D**) Robot-arm; (**E**) Microcontroller unit for control; (**F**) Serial communication module—USB FIFO.

The current sensor can be moved by robot-arm. Robot arm is shown in Figure 11. In experiment, RBD-707MG servo motor is used for robot-arm. Servo motor specification are: Type: RBD-707MG; Size: 50 × 40 × 20 mm; Weight: 60 g; Torque: 7.8 kg/cm; Operation voltage: 6V; Resolution angle: 0.5°; Speed: 0.17 s/60°. Mobile robot and robot-arm for measurement include servo motors of each joint, motor driver, ARM cortex M4 microcontroller based (STM32F407) control system, FT2232H based USB interface, two CMOS camera, and mobile robot using iRobot create. A mobile robot is used instead of inspection robot. ARM Cortex M4 microcontroller was used for all of controls, which controls servo motors of manipulator, and reads remote sensed current values. However, image processing was carried out in microcomputer. The line position was sensed by two image cameras. The camera capturing processing was implemented Video lab tools in RAD Studio C++ builder. However, image processing of threshold, filtering, edge detection, shape detection, and removing abnormal object were implemented by OpenCV functions.

**Figure 12:**
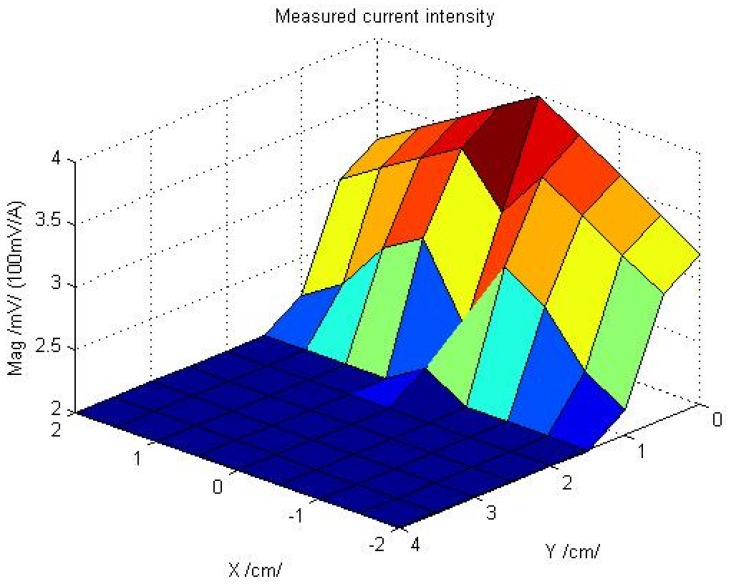
Remote current measurement using split core current sensor.

This experimental process was implemented by different positions of the remote current sensor. The current sensor was placed under the electric line by X and Y direction. In every position of the current sensor, the current intensity was measured. The measurement results are shown in Figure 12.

From the experimental result, the minimum permissible track distance should be around 5 mm. Therefore, in order to correct the current measurement, the minimum permissible track distance for the tracking algorithm is very important.

### 3.2. Simulation Results

In this paper, three simulation process was implemented. In order to implement simulation of the proposed overall algorithm, simulation of electrical line swing model, simulation of cylindrical manipulator model and simulation of line tracking algorithm are required. To describe swing trajectory of electrical conductor, parabolic function is used. In order to get measurement data with noise, Guassian white noise with 40 percent is added into distance data of between electrical conductor and proximity sensor. However, in cylindrical manipulator simulation, the above mentioned kinematics model is used. Furthermore, last simulation is carried out by the proposed overall tracking algorithm presented in Figure 8. In this model of the line swing, we assume that the swing trajectory of the electric line does not change due to wind speed and direction. All of the simulation models in this paper were carried out in MATLAB.

**Figure 13:**
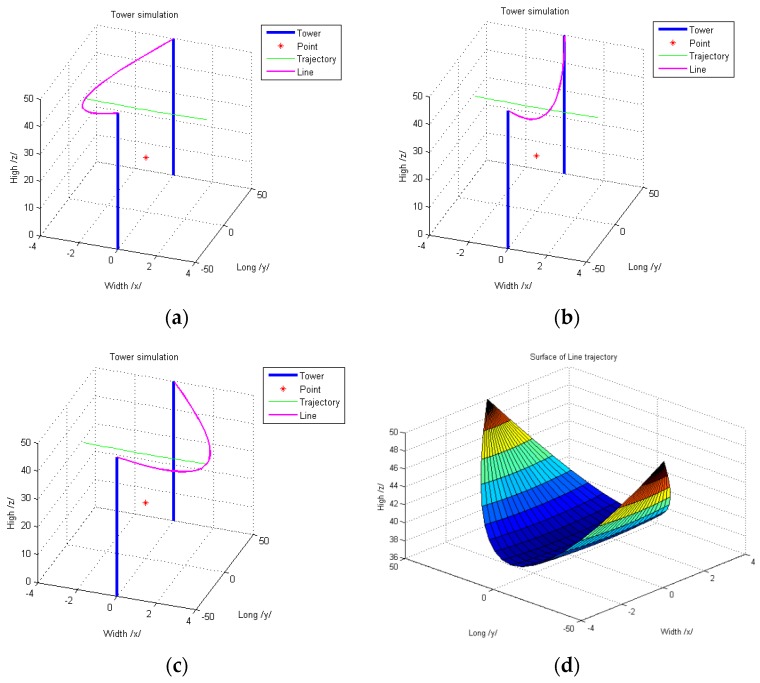
Simulation results of the electric line swing: (**a**) first scene of line swing; (**b**) second scene of line swing; (**c**) third scene of line swing; (**d**) surface of line trajectory.

Several important parameters should be inserted in the electric line swing model. These are tower height, distance between the two towers, electric line sag, wind speed and the remote current sensor position coordinates. The tower height and the distance between towers depends on electric line construction. However, electric line sag depends on the type of electric transmission wire, environmental conditions, and the climate. Simulation results of this model are shown in Figure 13. It shows some scenes of the line swing and the swing surface of the line trajectory. These results are required in the simulation model for the new tracking algorithm.

**Figure 14:**
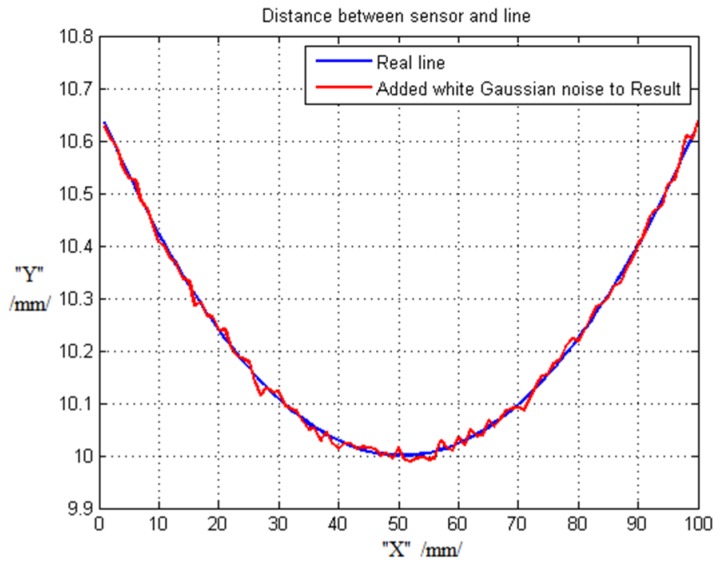
Electric line trajectory: Real trajectory of line swing and trajectory of line swing with Gaussian noise.

The next simulation result is the measurement of distances from the current sensor to the electric line during the electric line swing, which are determined in the current sensor constant position. However, during determination, the electric line position is moved using the line swing model. Simulation results are shown in Figure 14. In addition, in order to consider real environmental distance measurement data, Gaussian white noise with 40 percent was added into the determined distance data. This result is shown in Figure 14. The above process is related in the generated error of proximity sensors. If this measured distance data with error is used, the movement of line tracking will contain a little bit of vibration. This noise is represented by the red line in Figure 14.

**Figure 15:**
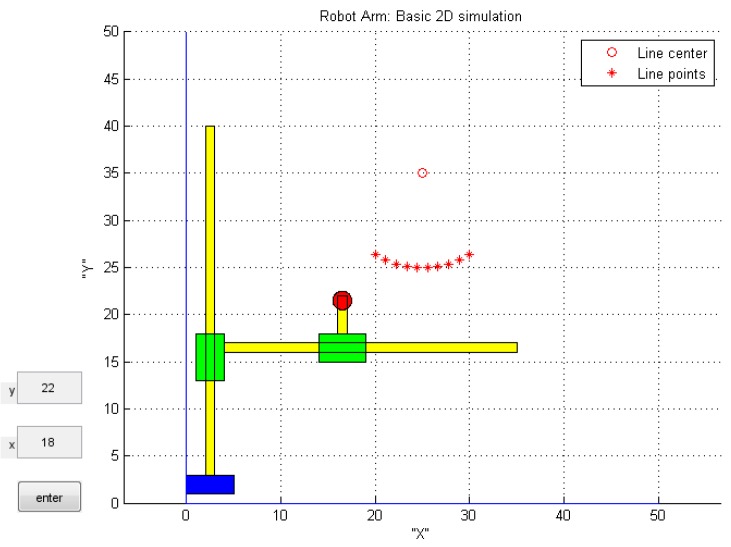
Simulation result of robot manipulator.

In order to check the tracking algorithm, there is a necessary model of the robot manipulator movement. In this paper, the model of the robot manipulator was implemented. The robot manipulator can move to the inserted coordinate in 2D space. One movement result of the manipulator is shown in Figure 15.

**Figure 16:**
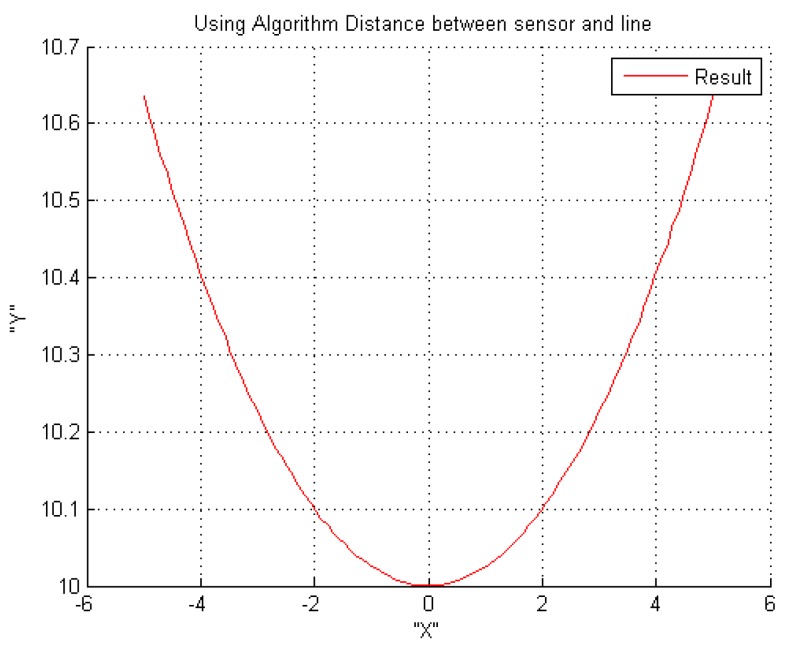
Traveling result of robot manipulator using the new algorithm.

The final simulation model is of the new tracking algorithm. This model consists of the above mentioned new algorithm in Section 2. Figure 16 shows the final tracking result, which represents the current sensor trajectory on the robot manipulator. From the results, we can see that the manipulator movement is smooth, and the Y direction errors are corrected.

## 4. Conclusions

In this paper, an inspection robot with a new tracking method was proposed for detecting illegal electricity usage. Experimental and simulation results of the proposed method were consistent with the desired descriptions. From the results of the simulation processes, the new proposed algorithm was useful, and the tracking performance could be increased.

In the future, it is necessary to implement a real experiment of the robot manipulator with the proposed algorithm, and to compare this with the simulation results. In addition, we need to consider the selection issue of a robot-arm, its type, and control hardware for a real experiment. These processes will be covered in our future research.
